# Ruyiping formula inhibits metastasis via the microRNA-134-SLUG axis in breast cancer

**DOI:** 10.1186/s12906-021-03365-4

**Published:** 2021-07-05

**Authors:** Ziwei Jiang, Lixia Pei, Ying Xie, Qun Ye, Xiaoqiang Liang, Yiyi Ye, Sheng Liu

**Affiliations:** Institute of Chinese Traditional Surgery, LongHua Hospital Affiliated to Shanghai University of Traditional Chinese Medicine, 725 Wanpingnan Road, Shanghai, 200032 China

**Keywords:** *Breast cancer*, *Metastasis*, *Ruyiping formula*, *miRNA-134*, *SLUG*, Epithelial–mesenchymal transition

## Abstract

**Background:**

Metastasis is the leading cause of death among breast cancer patients. MicroRNA-134 has been reported to have a tumor-suppressive role in breast cancer. Ruyiping (RYP), a traditional Chinese formula, has been shown with the ability to reduce breast cancer metastasis in pre-clinical studies. This present study was designed to examine whether miR-134 was involved in RYP-inhibited breast cancer metastasis.

**Methods:**

The expression of SLUG, E-Cadherin, N-Cadherin and miR-134 in MDA-MB-231 and 4 T1 cells treated with RYP or vehicle control were determined by quantitative realtime-PCR and western blot. Invasiveness determined by transwell assay as well as SLUG gene expression determined by qPCR were detected in cells transfected with chemically synthesized miR-134 mimics or inhibitors. BALB/c mice were injected with 4 T1 cells orthotopically and fed with RYP through gavage. Breast tumor growth, metastasis and tumor expression of EMT markers were detected.

**Results:**

Compared with the control, Ruyiping formula significantly inhibited SLUG-regulated breast cancer cells invasion. MiR-134 was induced by RYP in vitro and in vivo and was able to suppress SLUG by targeting its 3’UTR. RYP suppressed SLUG expression and cell invasion through miR-134. In 4 T1 tumor-bearing mice, RYP significantly inhibited 4 T1 tumor growth and lung metastasis, increased the levels of miR-134 and epithelial marker while decreased the levels of SLUG and mesenchymal marker.

**Conclusion:**

Our data uncovered that *Ruyiping* formula exerts an anti-metastatic activity against breast cancer cells by regulating SLUG through miR-134. MiR-134-SLUG axis might be a promising strategy in breast cancer therapy.

**Supplementary Information:**

The online version contains supplementary material available at 10.1186/s12906-021-03365-4.

## Background

Breast cancer is a common women malignancy in the world [1] which accounts for 367,900 new diagnosis and 97,972 deaths of Chinese female in 2018 [2]. Although substantial advances in modern therapy such as early diagnosis, chemotherapy and neoadjuvant treatment have improved survival rate of breast cancer, the mortality rate is still high. Metastasis is the major cause leading to treatment failure and patients fatality [3]. Therefore, it is extremely important to understand the molecular mechanisms of the metastasis of breast cancer and to screen for new, specific and sensitive targets for therapy.

Traditional Chinese herbal medicines have been widely used in clinical practice [4, 5]. Ruyiping Formula (RYP) is a traditional Chinese medicine (TCM) empirical prescription developed in Longhua Hospital, China. Previous pre-clinical studies have shown that RYP is effective in preventing breast cancer metastasis by protecting the microvascular integrity and suppressing tumor cell growth and migration [6, 7]. It is composed of *Iphigenia indica Kunth* (Shancigu), *Nidus Vespae* (Lufengfang), *Curcuma phaeocaulis Valeton* (Ezhu), raw seeds of *Coix lacryma-jobi L.* (Shengyiyiren) 12 g and *Akebiae Fructus* (Bayuezha). Some components of RYP had been shown with anti-tumor effects, such as Curcumin in *Curcuma zedoaria* and caffeic acid phenethyl ester (CAPE) derived from *Nidus Vespae* [8, 9]. However, the specific mechanisms of how RYP inhibits metastasis still remains to be explored.

Metastasis is responsible for more than 90% of cancer-related death [10]. EMT plays the major role in cancer metastasis [11]. Multiple cytokines, growth factors and transcription factors have been shown to participate in the EMT progression. SNAI2 (SLUG), a member of zinc-finger transcription factor Snail superfamily, is reported as a marker of malignancy and a key factor in EMT progression [12]. SLUG can be induced by TGF-β in cancer cells and then directly binds to the promoter region of E-Cadherin gene to suppress its transcription, thus promotes cancer cell migration [11, 13]. Therefore, SLUG might be an attractive target for therapeutic modulation of invasiveness in human cancer treatments. However, the molecular mechanism regulating the expression of SLUG is not clearly elucidated.

MicroRNAs are important in translational or post-transcriptional regulation. They are small noncoding RNAs about 20–25 nt in length and bind to the 3′-untranslated region (UTR) of target mRNAs to exert their inhibition function [14]. Numerous studies have demonstrated that miRNAs participate in tumorigenesis and carcinogenesis. MiR-134, first identified as a brain-specific miRNA [15], was recently reported significantly downregulated in breast cancer cells and reversely correlated with cell proliferation, lymph node metastasis, TNM stages and reduced cell differentiation [16, 17]. Moreover, it was reported to suppress EMT in multiple cancer types [16, 18].

The important roles of SLUG and miR-134 in EMT and tumor progression prompt us to hypothesize that they may be involved in RYP-inhibited breast cancer metastasis. In this study, we found that RYP induced miR-134 and suppressed SLUG expression in breast cancer both in vitro and in vivo. In the meantime, miR-134 suppressed SLUG 3’UTR activity through its potential binding site. We also demonstrated the importance of miR-134 and SLUG in the RYP-suppressed cancer cell invasion in vitro. Therefore, suppression of SLUG by induction of miR-134 might be the essential mechanism in the RYP-suppressed EMT, a key process involved in RYP-suppressed lung metastasis which was observed in a xenograft model.

## Methods

### Materials

The composition of Ruyiping formula were shown in Table 1 and all ingredients were purchased from Shanghai Kang Qiao Chinese Cut Crude Drug Co.,Ltd. with fixed origin and quality control standards. Morphological, microscopic, and phytochemical identification were performed by Professor Zhi-Li Zhao from the Department of Pharmacognosy, Shanghai University of Traditional Chinese Medicine. According to the Pharmacopoeia of the People’s Republic of China (2015 edition), the rhizome of *Curcuma phaeocaulis Valeton*, and the mature fruits of *Akebiae Fructus* were boiled, then air-dried before storage. Pseudobulb of *Iphigenia indica Kunth*, nest of *Vespae Nidus* and raw seeds of *Coix lacryma-jobi L.* were cleansed and air-dried before submitted to storage.. The herbarium voucher specimens were deposited in the Institute of Chinese Traditional Surgery, Longhua Hospital affiliated to Shanghai University of Traditional Chinese Medicine, with voucher numbers SCG-1907033 (*Iphigenia indica Kunth*), FF-1907034 (*Nidus Vespae*), EZ-1907035 (*Curcuma phaeocaulis Valeton*), SYY-1907036 (raw seeds of *Coix lacryma-jobi L.*) and BYZ-1907037 (*Akebiae Fructus*).

**Table 1:** The composition of Ruyiping formula

Chinese Name	Latin Name	Origin
Ezhu	*Curcuma phaeocaulis Valeton*	Guangxi
Shancigu	*Iphigenia indica Kunth*	Guizhou
Fengfang	*Nidus Vespae*	Shandong
Bayuezha	*Akebiae Fructus*	Shandong
Yiyiren	*Coix lacryma-jobi L.*	Guizhou

RPMI-1640 medium and fetal bovine serum were obtained from Gibco (Grand Island, New York, USA). Antibodies against SLUG (A1057), Actin (AC004), E-Cadherin (A11492), N-Cadherin (A0433) were obtained from ABclonal, Inc. (Wuhan, China). SLUG siRNAs, hsa-microRNA-134 and mmu-microRNA-134 mimics and inhibitors were purchased from Biotend Biotechnologies Co., Ltd. (Shanghai, China).

### Preparation of RYP decoction

RYP decoction was prepared according to previous method [6] with minor modifications. Twelve-gram *Iphigenia indica Kunth* (Shancigu), 12 g *Nidus Vespae* (Lufengfang), 12 g *Curcuma phaeocaulis Valeton* (Ezhu), 12 g raw seeds of *Coix lacryma-jobi L.* (Shengyiyiren) and 9 g *Akebiae Fructus* (Bayuezha) were mixed and soaked in 55% ethanol (1:10, w/v) for 4 h and then refluxed for 1.5 h. After filtering, the decoction was collected and the residues were refluxed again with 55% ethanol (1:8, w/v) for 1 h. Two parts of decoctions were combined and concentrated, and the concentrated extract was freeze-dried to obtain the RYP extract. The extract was dissolved in dimethyl sulfoxide and diluted with PBS.

### Cell culture and stimulation

Human breast carcinoma cell line MDA-MB-231 and mouse mammary carcinoma cell line 4 T1 were purchased from the Cell Bank of Type Culture Collection of the Chinese Academy of Sciences (Shanghai, China). MDA-MB-231 cells were cultured in DMEM medium and 4 T1 cells were cultured in RPMI-1640 medium with 10% fetal bovine serum at 37 °C in a 5% CO_2_ humidified chamber. Cells were grown to about 70% confluence before the RYP was used to stimulate cells for 24 h. Cells were collected for RNA isolation or protein extraction.

### Animals and experimental pre-metastatic model

BALB/c mice were purchased from SLAC Laboratory Animal Co. Ltd., (Shanghai, China) and raised in individual cages under specific-pathogen-free (SPF) level in the animal facility of Longhua Hospital affiliate to Shanghai University of Traditional Chinese Medicine. Mice were maintained in a temperature-controlled facility with a strict 12 h light/dark cycles and were given free access to food and water. All animal experiments were approved by the Institutional Animal Care and Use Committee (IACUC) of Longhua Hospital affiliated to Shanghai University of Traditional Chinese Medicine. All the surgeries were performed under anesthesia and all the efforts were made to minimize suffering.

The 4 T1 cells were digested and washed two times by cold PBS. The suspension concentration was adjusted to 2 × 10^6^ cells/mL. Except for the control group, each mouse was injected 50 μL into the fourth mammary fat pads on the right side. RYP extract was gavage administration was given daily for day 8 to day 35. After anaesthetized with isoflurane, mice were euthanized by cervical dislocation. The tumor growth was photographed and calculated by the formula *V = (W)*^*2*^ *× L/2.* Metastatic nodules in lung were stained with Bouin’s solution. Relative gene expression were detected by real-time PCR or western blot.

### SLUG overexpression

The open reading frames (ORFs) of mouse SLUG were amplified from cDNAs using gene-specific primers. Primers used in present study were shown in Table S1. After subcloning and sequencing, the correct gene fragments were inserted into pCMV-Tag2b. pCMV-Tag2b-SLUG plasmids were then transfected into 4 T1 cells by using Lipofectamine 2000 (Invitrogen). After overnight culture, the transfected cells were treated with RYP. All the information of these DNA sequences was from NCBI database.

### Real-time quantitative RT-PCR

Total RNA was extracted from mouse primary tumors or cells using TRIzol reagent (Life Technologies) following the manufacturer’s introduction. Complementary DNA was synthesized using cDNA Reverse Transcriptase kit (Q-111-02, Vazyme). Quantitative real-time (RT)-PCR-specific primers were designed and validated by NCBI Primer designing tool (https://www.ncbi.nlm.nih.gov/tools/primer-blast/) and all primers are shown in Table S1. Quantitative RT-PCR was performed in triplicate using SYBR green master mix (Vazyme) on a QuantStudio 3 System (Applied Biosystem). The samples with low yield of RNA were pre-determined and excluded. Quantitative analysis was performed using 2^-ΔΔ^Ct method for quantification of the relative mRNA expression.

### Immunoblot and immunohistochemistry

2 mm^3^ tumor samples or treated cells were lysed with RIPA buffer (pH 7.4) containing protease inhibitor cocktail (Roche). 20 μg of total protein was used for immunoblot. Samples were separated with SDS-PAGE and then transferred to nitrocellulose membranes followed by probing with indicated SLUG, E-Cadherin, N-Cadherin and Actin antibodies. The blots were visualized with ECL Enhanced Kit (Abclonal) on a ChemiDoc Touch Imaging System (Bio-Rad). The densitometry of all the bands was analyzed by Image J and normalized to GAPDH.

For immunostaining, five micrometers of formalin-fixed, paraffin-embedded tissue sections were mounted on glass slides. The sections were then dewaxed and subsequent pretreated with antigen retrieval solution. The sections were stained with anti-mouse SLUG antibody (Abclonal) overnight, then incubated with the HRP-labeled goat anti-mouse IgG (H + L) antibody (1:50 dilution, Beyotime Biotechnology) for 50 min. After DAB staining and hematoxylin counterstaining, sections were dehydrated with graded alcohol, hyalinized in xylene, and finally sealed with neutral gum. Under the microscope, the positive cells were stained brown.

### Invasion assay

24-well transwell plates equipped with the 8 μm pore in polyethylene terephthalate membranes (Corning Life Sciences, NY, USA) were used for detection. Matrigel (356,234, Corning Life Sciences) was diluted (1:30) with pre-cooled serum-free medium and added to the pre-cooled filter to form a thin gel layer. After 24 h treatment of RYP, cells were harvested and diluted to 1 × 10^6^ cells/mL in serum-free medium. 100 μL suspension was seeded into the upper chamber. The lower chamber was filled with.

600 μL of medium with 0.5% FBS. After 24 h incubation, the cells on the upper surface of the filter were removed by a cotton swab. Cells that invaded to the lower surface were stained with crystal violet and counted by IX71 microscope (Olympus).

### Target prediction and luciferase reporter assay

The SLUG 3’UTR were amplified with specific primers and cloned into pGL3-Control vector with a luciferase reporter (Promega). miR-134 binding sites were predicted by TargetScan (http://www.targetscan.org/), miRDB (http://www.mirdb.org/) and miRWalk (http://mirwalk.umm.uni-heidelberg.de/) database. Reporter plasmid carrying truncations and mutations at the 127 to 133 site (Fig. 3C) were generated by overlap PCR. The primers used were listed in Table S1. To test the activity of SLUG 3’UTR, 4 T1 cells were co-transfected with pGL3-SLUG 3’UTR-Luc plasmids and mmu-miR-134 mimics or inhibitors using Lipofectamine 2000 (Invitrogen). Twenty-four hours post transfection, the cells were collected and luciferase activity was measured on LUMIstar OPTIMA microplate reader (BMG LABTECH) by using the Dual-Luciferase Reporter Assay system (Promega).

### Statistical analysis

All data are present as mean ± s.e.m. We did analyses of multiple groups by one-way or two-way ANOVA with Bonferroni post test of GraphPad Prism Version 6. For all statistical tests, we considered *P* values < 0.05 to be statistically significant.

## Results

### RYP inhibits invasion through suppressing SLUG expression

**Fig. 1:**
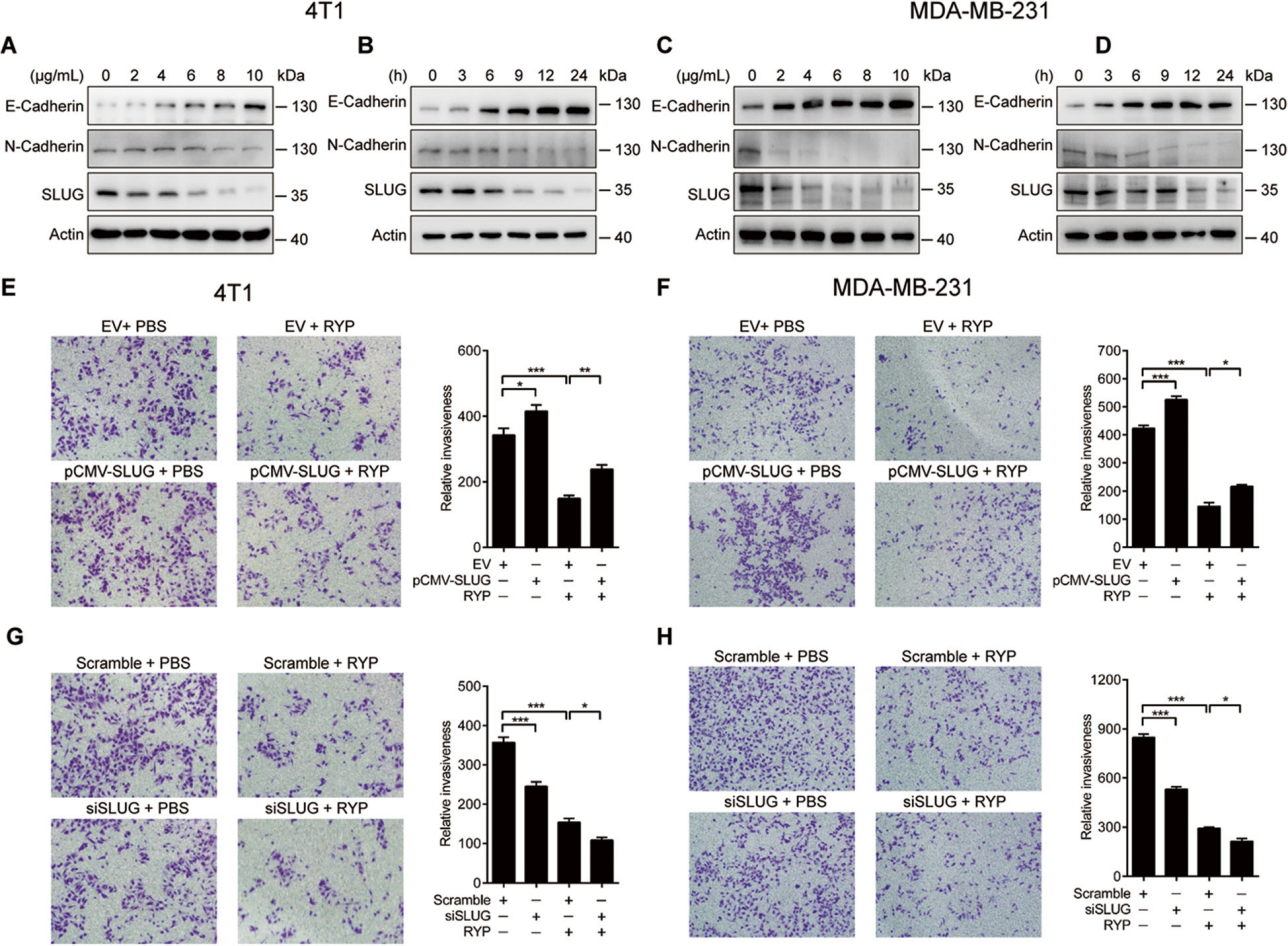
Ruyiping inhibits breast cancer cell migration by suppression of SLUG expression. E-Cadherin, N-Cadherin or SLUG production in 4 T1 (**A** and **B**) and MDA-MB-231 (**C** and **D**) cells stimulated with different dose of RYP (**A** and **C**) for 24 h, 10 μg/mL RYP (**B** and **D**) for indicated times. Transwell cell invasion assay of 4 T1 (**E** and **G**) and MDA-MB-231 (**F** and **H**) cells after SLUG overexpression (**E** and **F**) or silencing (**G** and **H**). Quantification of migrated cells were shown in the right figures. *P < 0.05, **P < 0.01, and ***P < 0.001. *P*-values were determined by one-way analysis of variance. Data are mean ± standard error of the mean and are representative of three independent experiments

Previous studies showed that RYP inhibits breast cancer lung metastasis. Since EMT play important roles in promoting migration, we thereby examined EMT genes expression in MDA-MB-231 and 4 T1 breast cancer cells after RYP treatment. Compared to the control group, RYP significantly decreased N-Cadherin expression and increased E-Cadherin expression in a dose- and time-dependent manner (Fig. 1A-D). Given that SLUG can bind to E-Cadherin promoter to inhibit its transcription, we tested whether RYP can regulate SLUG expression. Interestingly, SLUG was also inhibited in RYP–treated breast cancer cells (Fig. 1A-D). We then confirmed the role of SLUG in RYP-regulated invasion by invasion assay using transwell. In both MDA-MB-231 and 4 T1 breast cancer cells, RYP reduced breast cancer cells invasion through the Matrigel. Overexpression of SLUG reversed this RYP-induced suppression of invasion (Fig. 1E-F) while specific SLUG siRNA knockdown enhanced it (Fig. 1G-H). These data demonstrated that RYP inhibits breast cancer cells invasion through suppression of SLUG expression.

### RYP induces miR-134 to decrease SLUG translation

**Fig. 2:**
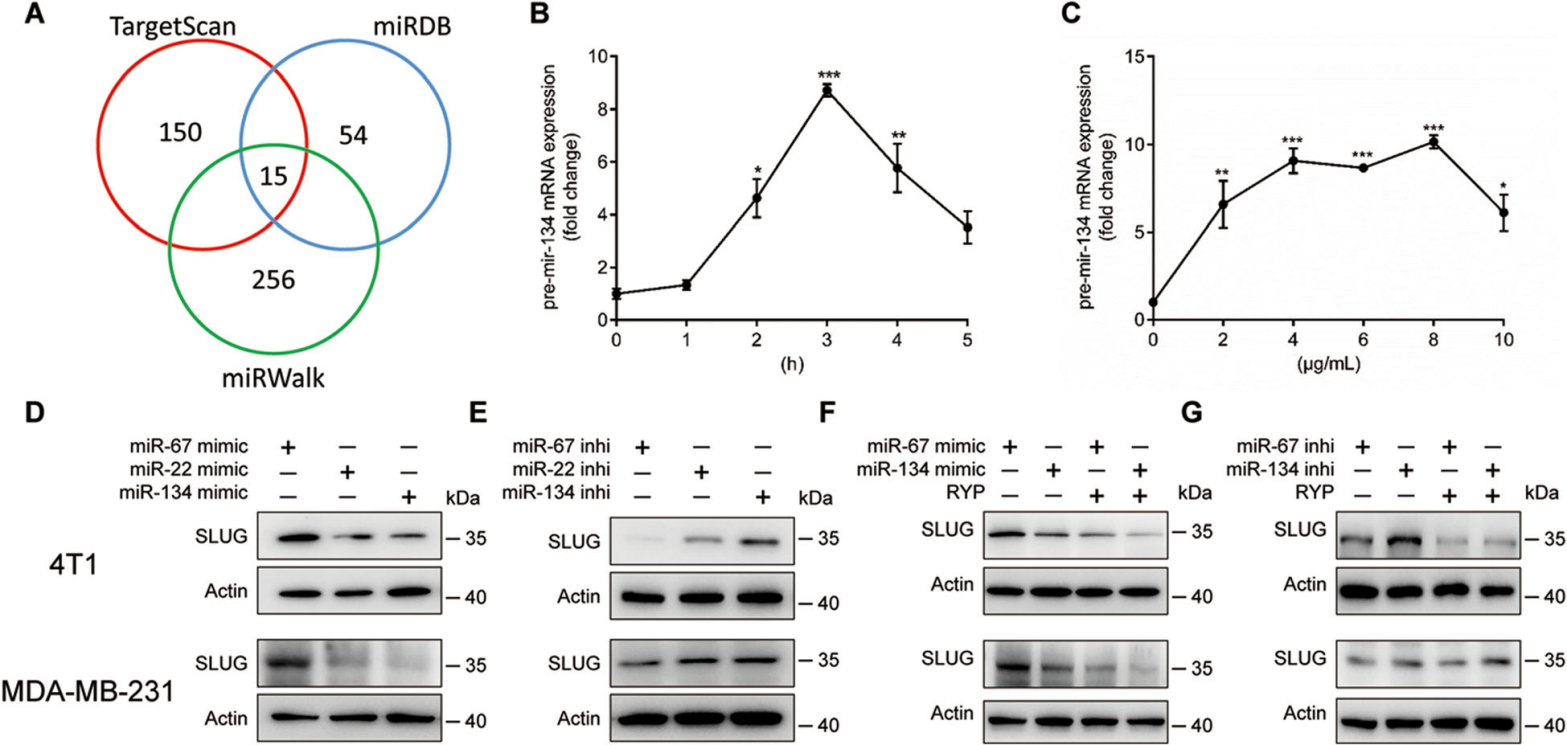
Ruyiping inhibits SLUG gene expression through miR-134 induction. Potential target miRNAs of SLUG 3’UTR were co predicted with the TargetScan, miRWalk and miRDB databases (**A**). Pre-miR-134 in 4 T1 cells stimulated with different dose of RYP (**B**) for 24 h, 10 μg/mL RYP (**C**) for indicated times. SLUG production in MDA-MB-231 and 4 T1 cells were detected after transfected with miR-22, miR-134 chemically synthesized mimics (**D**) or inhibitors (**E**) or treated with RYP (**F** and **G**). *P < 0.05, **P < 0.01, and ***P < 0.001. *P*-values were determined by one-way analysis of variance. Data are mean ± standard error of the mean and are representative of three independent experiments

MicroRNAs are important in suppression of gene expression. To further explore the mechanism how RYP inhibits SLUG expression, we used TargetScan (http://www.targetscan.org/), miRDB (http://www.mirdb.org/) and miRWalk (http://mirwalk.umm.uni-heidelberg.de/) databases to predict possible miRNAs involved in regulating SLUG expression There are 15 miRNAs with high scores in all three databases (Fig. 2A). Eight of them were not reported to regulate SLUG and therefore drew our interest to test whether they are new candidates of SLUG regulator. We further searched their reported relationship with breast cancer and found miR-92a, miR-25 and miR-134 to be most studied in breast cancer. Within them, miR-134 has the highest score in all three databases and was our focus in this study.

Therefore we detected pre-miR-134 expression in RYP-treated 4 T1 cells by qPCR. RYP induces pre-miR-134 expression in breast cancer cells in a time- and dose-dependent manner (Fig. 2B and C). To further confirm the role of miR-134, we transfected chemically synthesized miRNA mimics and inhibitors into MDA-MB-231 and 4 T1 cells. SLUG-irrelevant miRNA, miR-67, was used as a negative control. MiR-22 was used as a positive control, which has been reported before as a negative regulator of SLUG. MiR-134 mimics significantly decreased SLUG protein level (Fig. 2D) while miR-134 inhibitors increased SLUG expression (Fig. 2E) which were similar with miR-22 group. Moreover, we used RYP to treat miRNAs-transfected 4 T1 and MDA-MB-231 cells. MiR-134 mimics transfection enhanced RYP-induced SLUG reduction (Fig. 2F) while the miR-134 inhibitor reversed RYP’s function and increased SLUG expression (Fig. 2G). These results demonstrated that RYP inhibits SLUG gene expression through induction of miR-134.

### SLUG is a downstream target of miR-134

**Fig. 3:**
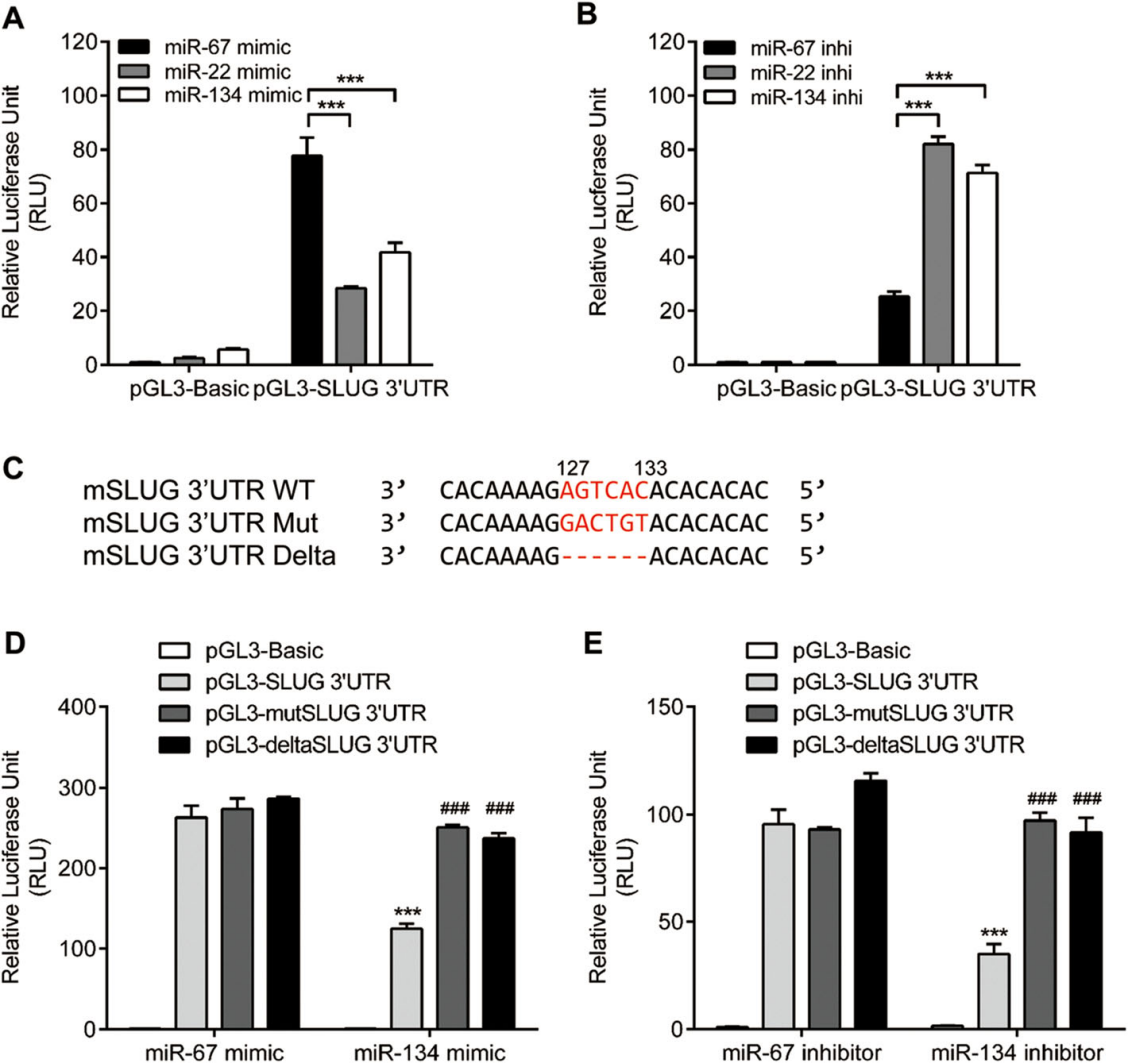
miR-134 binds to SLUG mRNA 3’UTR to suppress protein translation. Normalized luciferase activity in 4 T1 cells transfected with pGL3-control-SLUG 3’UTR vector and miR-22, miR-134 chemically synthesized mimics (**A**) or inhbitors (**B**). (**C**) The putative miR-134 binding sites; the wild-type, mutant (Mut) and deletion sequence of 3′-UTR of SLUG. Normalized luciferase activity in 4 T1 cells transfected with various reporter vector constructs with miR-67 or miR-134 mimics (**D**) or inhibitors (**E**). ***P < 0.001, ###*P* < 0.001. *P*-values were determined by one-way analysis of variance. Data are mean ± standard error of the mean and are representative of three independent experiments

We next explored the molecular mechanism by which miR-134 downregulated SLUG expression. 3’UTR region of mRNA is usually where miRNA binds to inhibit the translation. We first co-transfected wild-type SLUG 3’UTR reporter vectors with miR-134 mimics or inhibitors and miR-22 was used as a positive control. Compared to the control group, the reporter luciferase activity with a wild-type SLUG 3’UTR was significantly reduced by miR-22 or miR-134 mimics in 4 T1 cells (Fig. 3A). However, miR-22 or miR-134 inhibitors enhanced the luciferase activity (Fig. 3B). These results confirmed the inhibition of miR-134 in SLUG translation through its 3’UTR. To further confirm the potential binding site of miR-134 in the SLUG 3’UTR, we deleted or mutated it in the SLUG 3’UTR reporter plasmids (Fig. 3C). MiR-134 mimic diminished the reporter’s activity with the wild-type SLUG 3’UTR. However, after the potential binding site was deleted or mutated, miR-134 lose its ability to decrease the luciferase activity (Fig. 3D and E). These results suggested that miR-134 downregulates SLUG expression through directly binding to its 3’UTR potential binding site.

### RYP inhibits breast cancer cells invasion through miR-134

**Fig. 4:**
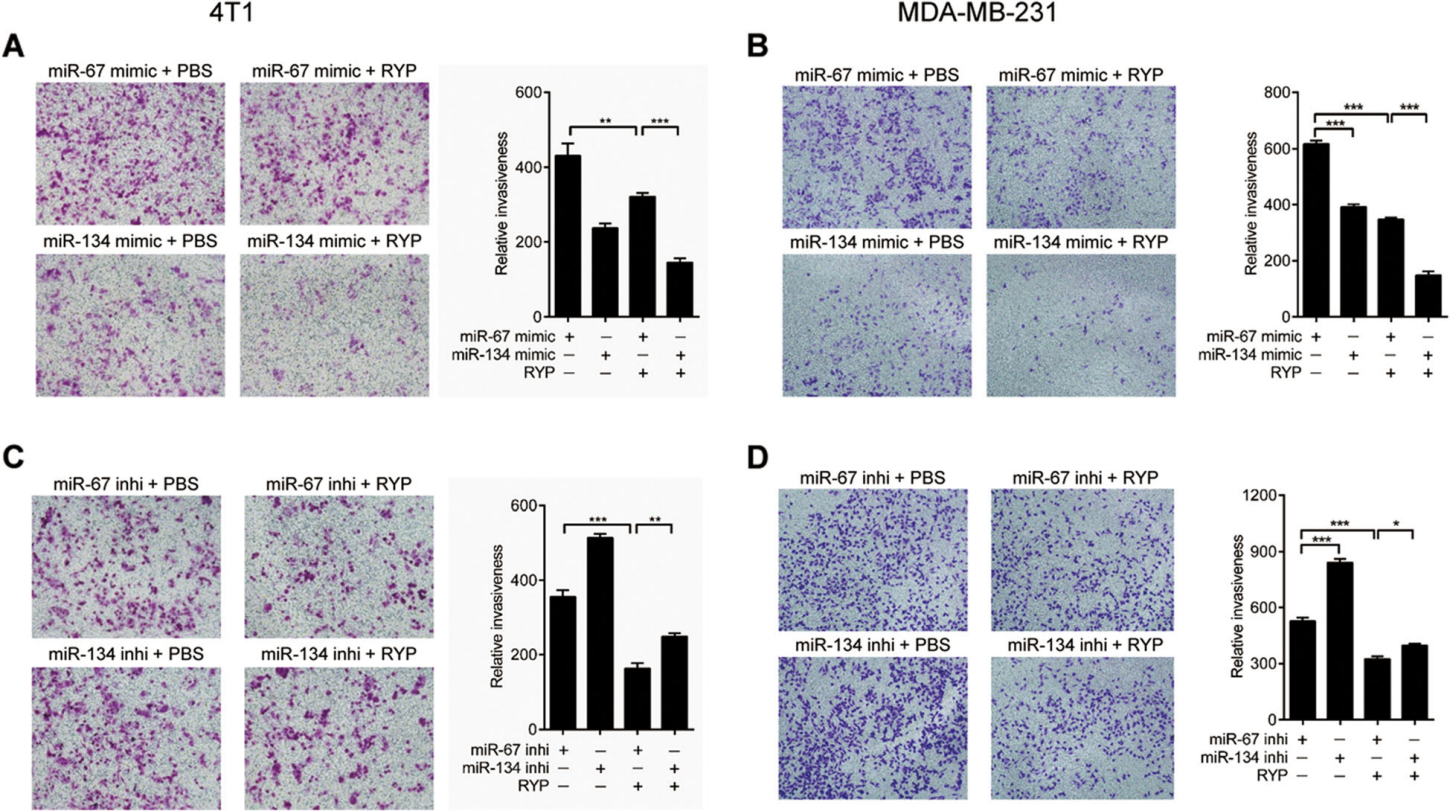
Ruyiping inhibits breast cancer cells migration through miR-134. Transwell cell invasion assay of MDA-MB-231 and 4 T1 cells stimulated with RYP after transfected miR-134 mimics (**A** and **B**) or inhibitors (**C** and **D**). Quantification of migrated cells were shown in the right figures. *P < 0.05, **P < 0.01, and ***P < 0.001. *P*-values were determined by one-way analysis of variance. Data are mean ± standard error of the mean and are representative of three independent experiments

To further confirm the role of miR-134 in RYP-inhibited breast cancer metastasis, we performed the invasion assay. RYP reduced MDA-MB-231 and 4 T1 cells invasion through the Matrigel as demonstrated before. MiR-134 mimics reduced breast cancer cells invasion and enhanced the reduction of invasion by RYP treatment (Fig. 4A and B). In contrast, miR-134 inhibitors increased invasion and aborted RYP’s reduction of invasion (Fig. 4C and D). These data demonstrated that miR-134 is an important microRNA involved in RYP-inhibited breast cancer cell invasion.

### The miR-134/SLUG axis is regulated by RYP in a tumor xenograft model

**Fig. 5:**
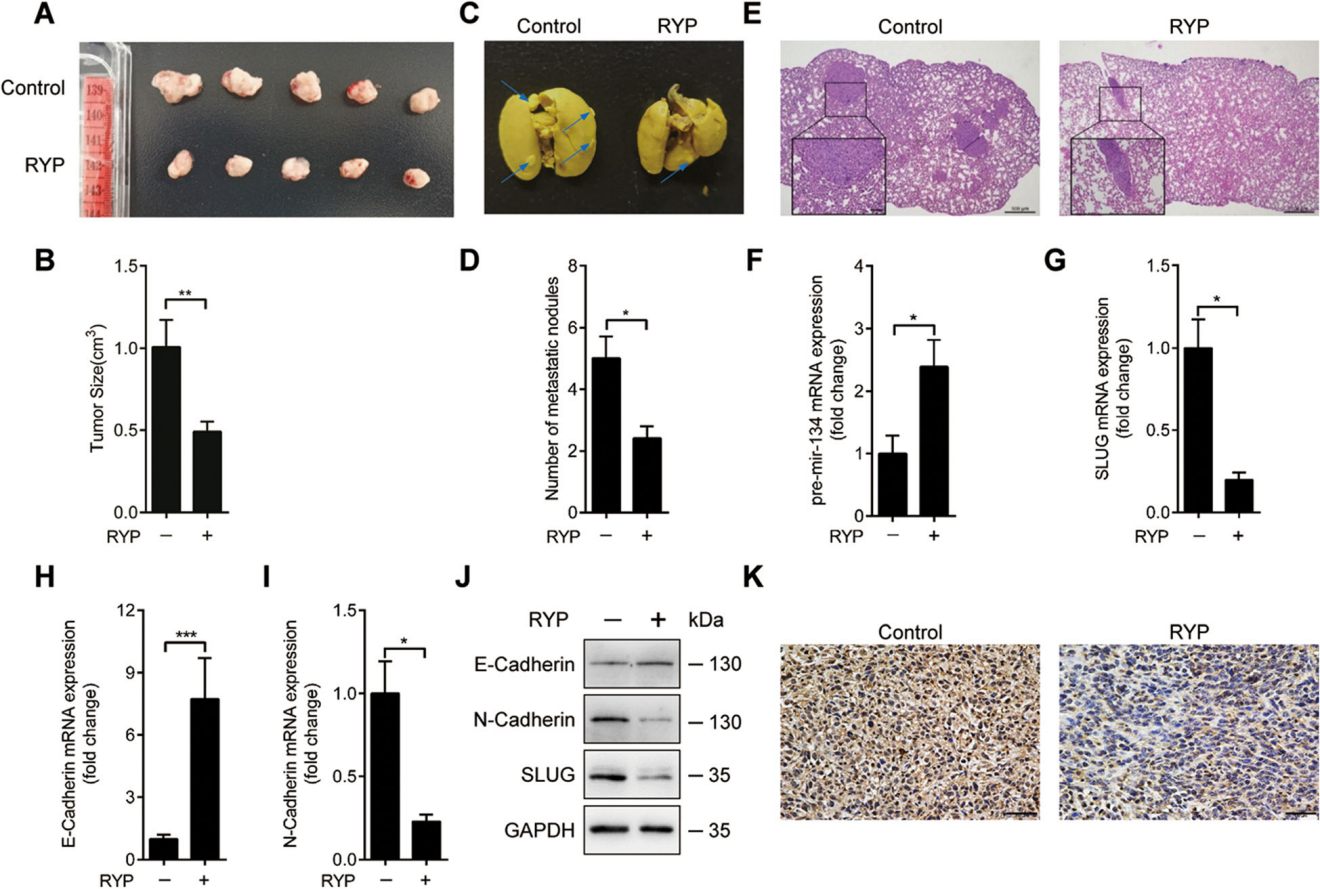
Ruyiping inhibits breast cancer metastasis in vivo. Mice transplanted with 4 T1 cells (*n* = 5) and gavage administration once a day with RYP. Xenograft tumor growth was photographed and calculated by the formula *V = (W)*^*2*^ *× L/2* (**A** and **B**)*.* Metastatic nodules in lung were stained with Bouin’s solution and counted (**C**-**E**). SLUG, E-Cadherin, N-Cadherin and pre-miR-134 expression in primary tumors were detected by real-time PCR (**F**-**I**) or western blot (**J**). (**E**) HE staining of lungs five weeks after injection of 4 T1 cells and treated with saline or RYP. Scale bars = 0.5 mm. Arrows designate region of 200*×* magnification shown in insets. Scale bars = 0.1 mm. (**K**) Immunohistochemical analysis of SLUG in primary tumors. Scale bars = 0.01 mm. *P < 0.05, **P < 0.01. *P*-values were determined by one-way analysis of variance. Data are mean ± standard error of the mean and are representative of three independent experiments

To validate the role of miR-134/SLUG axis in RYP-regulated breast cancer metastasis, we used a tumor xenograft model by injecting tumor cells into the mammary fat pads of BALB/c female mice. Tumor-bearing mice were detected to have extensive metastatic lesions in the lungs after 35 days, whereas RYP treatment significantly reduced the tumor size (Fig. 5A and B) and suppressed the lung metastasis (Fig. 5C, D and E), indicating that this formula has potential effect of tumor growth suppression. We further detected related gene expression in primary tumors. Consistent with the in vitro experiment results, RYP induced miR-134 and inhibited EMT related SLUG and N-cadherin gene expression while increased E-Cadherin expression (Fig. 5F-K). Altogether, these data supported that RYP induces miR-134 to inhibit SLUG expression and thus suppress EMT, which finally leads to suppression of tumor metastasis.

## Discussion

Chinese herb medicines (CHM) have a long history of clinical use in prevention and treatment of breast cancer [19]. Studies have demonstrated that CHM alleviates side effects and improves life quality of and treatment efficiency. For example, Wenshen Zhuanggu formula mitigates breast cancer bone metastasis through suppression of Jagged1/Notch and TGF-β1/Smads pathway [5, 20]. Yiqi formula inhibited the TNBC xenograft tumor growth by reducing p-EGFR and p-AKT1 [21]. Therefore, deeply understanding the mechanisms of CHM in tumor progression may provide new perspectives for cancer treatment.

Ruyiping formula (RYP) is established by clinical experiences of doctors and has been used in our hospital for more than 30 years with reduced recidivation and metastasis rates in breast cancer patients [22]. Previous pre-clinical studies have shown RYP in protecting the microvascular integrity and suppressing tumor cell growth and migration [6, 7]. Here we revealed a previously unreported function of RYP in regulating breast cancer metastasis via restoring E-Cadherin expression through inhibition of SLUG. The underlying mechanism involves the induction of miR-134, which abrogated SLUG translation by binding with its 3’UTR. Thus, the elucidation of the mechanism of RYP provides crucial information for understanding the traditional Chinese medicine (TCM). Further identification of RYP components and mechanism studies of them in breast cancer metastasis will be an interesting direction for our future studies.

Epithelial-mesenchymal transition (EMT) is a complicated biological process which contributes to tumor progression and metastasis [23]. About 70% of breast cancer patients with distant metastasis died within five years [24]. Multiple cytokines and growth factors participate in this process [25]. Main characteristics of EMT progression are decreased expression of E-Cadherin and increased expression of N-Cadherin. Epithelial cells lose cell polarity and cell junctions to obtain higher migration, extracellular matrix degrading and invasion abilities [23]. SLUG, a member of zinc finger Snail family, is an important EMT inducer in lung and breast cancer. As a transcriptional repressor, SLUG has been observed to bind to the promoter E-box site together with Lysine Specific Demethylase 1 (LSD1) to repress the expression of E-Cadherin transcription, and thus promote the cancer cells migration [26, 27]. In this study, RYP treatment on breast cancer cells significantly reduced expression of SLUG as well as cell invasiveness compared to the untreated group. SLUG overexpression reversed this effect by RYP, while SLUG-silencing in 4 T1 and MDA-MB-231 cells enhanced the RYP-induced suppression of cell invasiveness. These data were consistent with the previous observations that SLUG-induced EMT [28] and confirmed that RYP inhibits breast cancer cell invasion by reducing SLUG expression.

miRNAs are known to function through gene inhibition and have been reported as significant regulators in diverse human diseases, especially in cancers [29]. In order to identify new SLUG-regulating miRNAs, we used TargetScan, miRDB and miRWalk databases to predict miRNAs binding with SLUG 3’UTR and identified a non-reported SLUG-regulator miR-134, which has high scores in all three databases and has been studied in breast cancer. MiR-134 belongs to chromosome 14q32 miRNAs clusters and was first reported in dendritic spine development, hippocampal memory and synaptic plasticity [15, 30]. Recently it is found to be essential for human carcinogenesis, tumor cell proliferation and metastasis and significantly downregulated in breast cancer cells, reversely correlated with cell proliferation, lymph node metastasis, TNM stage and reduced cell differentiation [16, 17]. MiR-134 was also shown to increase breast cancer cells sensitivity to chemotherapy [31] and has been shown to inhibit EMT in multiple cancers, including lung cancer, glioma, breast cancer and colorectal cancer [17]. In our study, we observed that RYP induced miR-134 expression both in vivo and in vitro. The induction of miR-134 peaked at 3 h of RYP treatment while the suppression of SLUG started to be evident at 6 h, which is consistent with the hypothesis that RYP suppress SLUG through induction of miR-134. Using a 3’UTR luciferase reporter system, we demonstrated that miR-134 mimics inhibited the SLUG 3’UTR activity but had no effect when the binding site was mutated or deleted. In contrast, miR-134 inhibitors showed opposite effects. Therefore, we confirmed miR-134 to be a new regulator of SLUG gene through binding to its 3’UTR. Using miR-134 mimics and inhibitors we also confirmed that RYP inhibited SLUG expression and reduced cancer cell invasiveness through induction of miR-134.

**Fig. 6:**
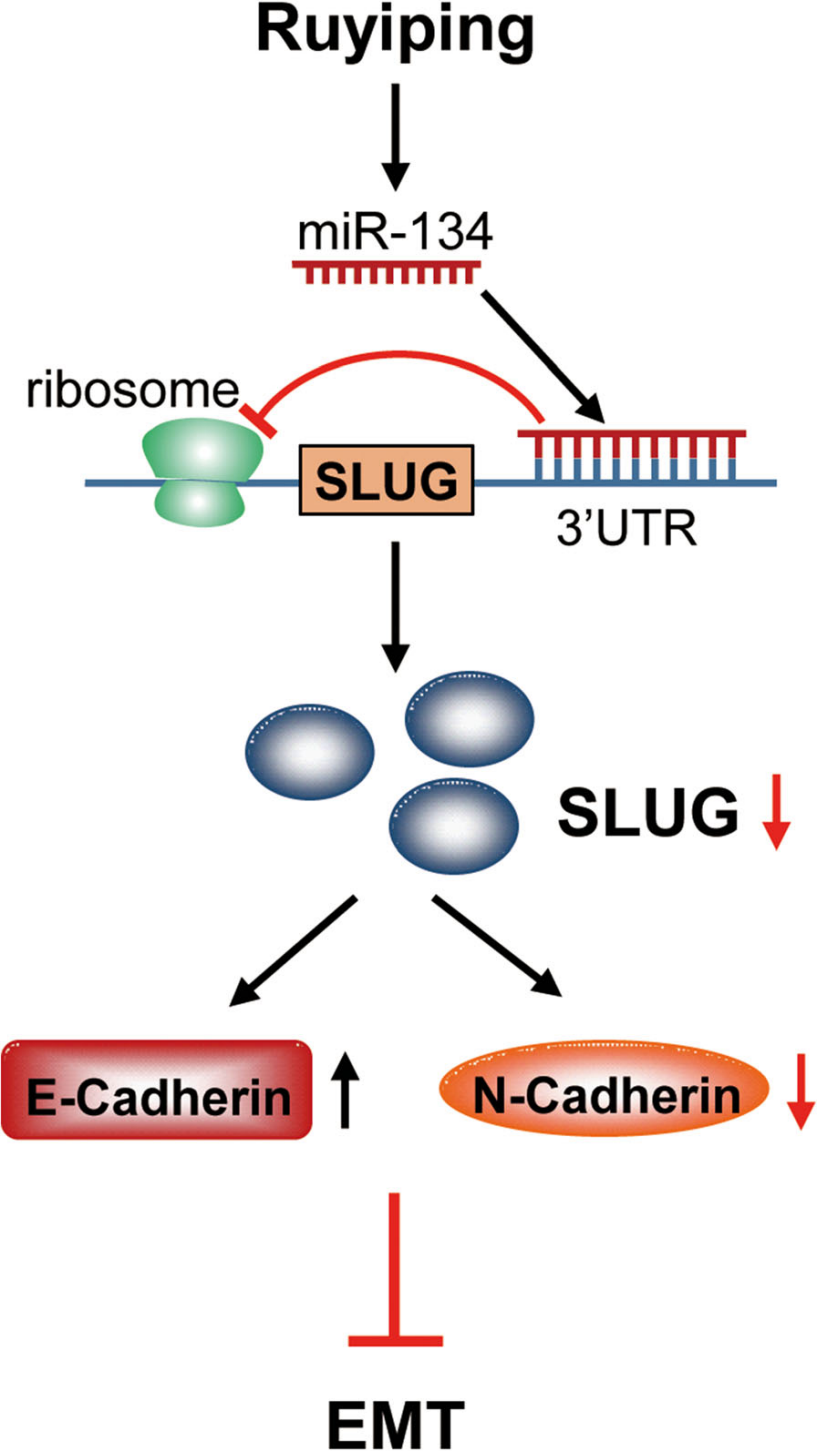
The molecular mechanism by which RYP inhibited the EMT. RYP inhibited SLUG through induction of miR-134, which interrupted SLUG translation by binding to its 3’UTR. Inhibition of SLUG resulted in increase of E-Cadherin and decrease of N-Cadherin, which caused a suppression of the EMT process

In a summary shown in Fig. 6, we proposed the following mechanism by which RYP inhibited the EMT and thus inhibited breast cancer growth and lung metastasis. RYP inhibited SLUG through induction of miR-134, which interrupted SLUG translation by binding to its 3’UTR. Inhibition of SLUG resulted in decrease of N-Cadherin and increase of E-Cadherin, which caused a suppression of the EMT process. The in vivo data from mouse model confirmed this mechanism. RYP not only increased miR-134 and decreased SLUG expression in the tumor, but also reduced EMT since E-Cadherin was increased and N-Cadherin was reduced.

Further studies are necessary to explore the tissue distribution and pharmacokinetic mechanism of bioactive compounds from RYP extract in animal models. They will facilitate in predicting various events related to the clinical efficacy and tolerability of this TCM formula against breast cancer metastases.

## Conclusions

In summary, these findings support our discovery that miR-134/SLUG axis is important in RYP-regulated tumor metastasis and the inhibition of the SLUG is due to the interruption of miR-134 at 3’UTR site. MiR-134 might be a potential therapeutic target in metastasis. Moreover, the delineation of the mechanism involved in the inhibition of SLUG expression provides insights into pathways contributing to Ruyiping clinical treatment of breast cancer.

## Supplementary Information

**Additional file 1.**

**Additional file 2.**

## Data Availability

The datasets used and/or analyzed during the current study are available from the corresponding author on reasonable request.

## Abbreviations

RYP: Ruyiping formula
SLUG: snail family transcriptional repressor 2
EMT: Epithelial–mesenchymal transition
3’UTR: 3′-untranslated region
TCM: traditional Chinese medicine;
CHB: Chinese herb medicines
miR-: micro RNA
