# Supplementary material for: Ruyiping formula inhibits metastasis via the microRNA-134-SLUG axis in breast cancer
Source: BMC Complement Med Ther. 2021 Jul 5;21:191. doi: 10.1186/s12906-021-03365-4 (PMC8258945; doi:10.1186/s12906-021-03365-4)
Supplement: Supplementary file 1 — Additional file 1. [file 12906_2021_3365_MOESM1_ESM.docx]

**Supplementary Table S1 siRNA oligonucleotides and primers**

| Mouse SLUG siRNA1 | sense | GCUCCUUCCUGGUCAAGAA |
| --- | --- | --- |
|  | anti-sense | UUCUUGACCAGGAAGGAGC |
| Mouse SLUG siRNA2 | sense | GGAUCACAGUGGUUCAGAA |
|  | anti-sense | UUCUGAACCACUGUGAUCC |
| Mouse SLUG siRNA3 | sense | GAAGUUUCAGUGCAAUUUA |
|  | anti-sense | UAAAUUGCACUGAAACUUC |
| Human SLUG siRNA1 | sense | CAUAUUCGGACCCACACAU |
|  | anti-sense | AUGUGUGGGUCCGAAUAUG |
| Human SLUG siRNA2 | sense | GUGACGCAAUCAAUGUUUA |
|  | anti-sense | UAAACAUUGAUUGCGUCAC |
| Human SLUG siRNA3 | sense | GUUGAUGAGUCAAUGUAAA |
|  |  | UUUACAUUGACUCAUCAAC |
| GAPDH | sense | CTTAGCCCCCCTGGCCAAG |
|  | anti-sense | TGGTCATGAGCCCTTCCACA |
| SLUG | sense | ACATTAGAACTCACACTGGGGA |
|  | anti-sense | ATTGCAGTGAGGGCAAGAGA |
| E-Cadherin | sense | GAAGGCTTGAGCACAACAGC |
|  | anti-sense | CCCTGATACGTGCTTGGGTT |
| N-Cadherin | sense | GGCAGATCACTACTATTGCCGT |
|  | anti-sense | GGCGGGATTCCATTGTCAGA |
| Pre-mmu-mir-134 | sense | AGGGTGTGTGACTGGTTGAC |
|  | anti-sense | GGGTTGGTGACTAGGTGGC |
| SLUG 3'UTR clone | sense | GGCTCTAGAGTGGCGCAACCAGTGTTTAC |
|  | anti-sense | CCGGTTAACTTTTTACTCAAAAATGTTTATTGTAAAA |
| SLUG 3'UTR mut clone | sense | TAGTATGTATAAAACCACAAAAGGACTGTACACACACACACACACACAC |
|  | anti-sense | GTGTGTGTGTGTGTGTGTGTACAGTCCTTTTGTGGTTTTATACATACTA |
| SLUG 3'UTR delta clone | sense | TAGTATGTATAAAACCACAAAAGACACACACACACACACACAC |
|  | anti-sense | GTGTGTGTGTGTGTGTGTGTCTTTTGTGGTTTTATACATACTA |
