# Supplementary material for: Ruyiping formula inhibits metastasis via the microRNA-134-SLUG axis in breast cancer
Source: BMC Complement Med Ther. 2021 Jul 5;21:191. doi: 10.1186/s12906-021-03365-4 (PMC8258945; doi:10.1186/s12906-021-03365-4)
Supplement: Supplementary file 2 — Additional file 2. [file 12906_2021_3365_MOESM2_ESM.docx]

**ARRIVE guidelines**

| Item |  | Section/ Paragraph |
| --- | --- | --- |
| Abstract | This aim of this study is to examine the effect of Ruyiping formula in preventing breast cancer metastasis.  Female BALB/c mice (8-week old) were injected in situ 4T1 cells (1×10^5^ cells per mouse) into the mammary fat pads and garaged with RYP. Breast tumor growth, metastasis and the expression of EMT markers were detected in vivo after 5-week experiment. Ruyiping formula significantly inhibited 4T1 tumor growth and lung metastasis, increase the level of miR-134 and decreased the levels of EMT genes in primary tumor sites. | Abstract |
| Background | 1. Ruyiping formula inhibits 4T1 breast cancer cells migration in Transwell assay. 2. Ruyiping formula induces miR-134 to suppression of SLUG protein translation. | Paragraph 1,4  Paragraph 2,3 |
| Objectives | This aim of this study is to examine the effect of Ruyiping formula in preventing breast cancer metastasis.  Given that Ruyiping formula inhibits critical EMT gene SLUG expression through induction of miR-134, we hypothesized that it can preventing breast cancer metastasis in mice tumor xenograft model | Paragraph 1-4 |
| Ethical statement | This study was approved by the Institutional Animal Care and Use Committee (IACUC) of Longhua Hospital affiliated to Shanghai University of Traditional Chinese Medicine. | Methods  Page 7 |
| Housing and husbandry | BALB/c mice were raised in individual cages under speciﬁc-pathogen-free (SPF) level in the animal facility of Longhua Hospital affiliate to Shanghai University of Traditional Chinese Medicine. Mice were maintained in a temperature-controlled facility with a strict 12h light/dark cycles and were given free access to food and water. | Methods  Page 7 |
| Animal care and monitoring | 1. Before mice were taken experimental treatment, Isoflurane was used to anaesthetize those mice. 2. No adverse events were found in this experiment. 3. During this experiment, mice weights were measured each weak and tumor size, lung metastasis nodules were measured or counted at the end of experiment. 4. After anaesthetized with Isoflurane, mice were euthanized by cervical dislocation. | Methods  Page 7 |
| Interpretation /scientific implications | 1. We found that control group of mice showed extensive metastatic lesions in the lung, whereas RYP-treated group significantly suppressed the lung metastasis. 2. After Ruyiping formula gavage, the tumor size became smaller than control group which indicates this formula has potential effect of tumor growth suppressor. 3. Ruyiping formula induced miR-134 and inhibits EMT related gene expression in primary tumors. 4. These results indicates the role of Ruyiping formula in mouse strain breast cancer cells which could further use nude mice and human strain breast cancer cells to confirm its role. | Paragraph 5 |
| Generalisability / translation | The results in this experiment showed great effects of Ruyiping formula in preventing 4T1 tumor-bearing mice which may further repeated in other tumor models such as human strain cancer cells bearing in nude mice. |  |
| Protocol registration | This study was approved by the Institutional Animal Care and Use Committee (IACUC) of Longhua Hospital affiliated to Shanghai University of Traditional Chinese Medicine. | Methods  Page 7 |
| Data access | The datasets used and/or analyzed during the current study are available from the corresponding author on reasonable request. | Declaration  Page 15 |
| Declaration of interests | There is no competing financial interests in this experiment. | Declaration  Page 15 |
